# Surfactant-Mediated Co-Existence of Single-Walled Carbon Nanotube Networks and Cellulose Nanocrystal Mesophases

**DOI:** 10.3390/nano11113059

**Published:** 2021-11-13

**Authors:** David Attia, Evgenee Yekymov, Yulia Shmidov, Yael Levi-Kalisman, Orit Mendelson, Ronit Bitton, Rachel Yerushalmi-Rozen

**Affiliations:** 1 Department of Chemical Engineering, Ben-Gurion University of the Negev, Beer-Sheva 84105, Israel; attiad@post.bgu.ac.il (D.A.); ekymov@post.bgu.ac.il (E.Y.); yuliashm@post.bgu.ac.il (Y.S.); rbitton@bgu.ac.il (R.B.); 2 The Center for Nanoscience and Nanotechnology, The Hebrew University of Jerusalem, Jerusalem 9190401, Israel; yael.kalisman@mail.huji.ac.il; 3 The Institute of Life Sciences, The Hebrew University of Jerusalem, Jerusalem 9190401, Israel; 4 Nuclear Research Center-Negev, Department of Chemistry, Beer-Sheva 84190, Israel; orit@post.bgu.ac.il; 5 The Ilse Katz Institute for Nanoscience and Technology, Ben-Gurion University of the Negev, Beer-Sheva 84105, Israel

**Keywords:** CNCs, SWNTs, chiral-nematic phase, co-existing networks

## Abstract

Hybrids comprising cellulose nanocrystals (CNCs) and percolated networks of single-walled carbon nanotubes (SWNTs) may serve for the casting of hybrid materials with improved optical, mechanical, electrical, and thermal properties. However, CNC-dispersed SWNTs are depleted from the chiral nematic (N*) phase and enrich the isotropic phase. Herein, we report that SWNTs dispersed by non-ionic surfactant or triblock copolymers are incorporated within the surfactant-mediated CNC mesophases. Small-angle X-ray measurements indicate that the nanostructure of the hybrid phases is only slightly modified by the presence of the surfactants, and the chiral nature of the N* phase is preserved. Cryo-TEM and Raman spectroscopy show that SWNTs networks with typical mesh size from hundreds of nanometers to microns are distributed equally between the two phases. We suggest that the adsorption of the surfactants or polymers mediates the interfacial interaction between the CNCs and SWNTs, enhancing the formation of co-existing meso-structures in the hybrid phases.

## 1. Introduction

Hybrid materials comprising single-walled carbon nanotubes (SWNTs) and liquid crystalline (LC) mesophase have been thoroughly investigated in the context of non-isotropic nano-composites [1]. Often, the presence of SWNTs modifies the structure and properties of the LC mesophase [2] and shifts the critical concentration (or temperature) at which the phase emerges [3]. There are rare examples in which the SWNTs reside within the LC phase at concentrations high enough to form percolated networks [4].

CNCs are rod-shaped nanocrystals with typical widths of few nanometers and lengths of some tens to hundreds of nanometers. When obtained via sulfuric acid hydrolysis of cellulose, they are negatively charged due to the presence of surface sulfate ester groups [5]. The CNCs form stable suspensions in aqueous media and above a critical volume fraction, φ*, and phases separate into a chiral nematic (N*) and optically isotropic (*I*) phases [6,7]. In these systems the nematic mesophase results from competition between the contribution of orientational entropy and the excluded volume to the free energy, as described by models based on mean-field excluded volume potential in a thermal solvent by Onsager and Flory [8,9,10]. Electrostatic effects are found to shift the phase diagram but preserve the overall behavior, as observed experimentally and rationalized by Stroobants, Lekkerkerker and Odijk (SLO theory) [11].

A steady rotation of the director in the N* phase of suspended CNCs results in helical modulation characterized by a cholesteric pitch, *P*, typically ranging from 3 to 100 µm [7]. When observed between crossed polarizers, the birefringent phase exhibits iridescent colors and a typical extinction pattern that results from the rotation of the pitch. The latter is known as the “fingerprint pattern”.

CNCs are known to disperse SWNTs in dilute suspensions, probably via interactions with the π electrons of the SWNTs. While the CNC-dispersed SWNTs are excluded from the N* phase [12], it was found that it is possible to utilize evaporation induced self-assembly (EISA) of CNCs suspensions [13,14,15,16,17] for preparation of thin dried films of CNC–SWNT hybrids. The EISA process is initiated from CNC suspensions in the isotropic phase (φ < φ*) [18] and the SWNTs are trapped in the drying suspension. The balance of intermolecular interactions in liquid mixtures of CNCs and SWNTs, and in particular the question of what drives the exclusion of SWNTs from the N*, are not yet well understood.

Studies of nanometric inclusions embedded in CNC LC phases envision the incorporation of nanoparticles into the N* phase as a size-selective process [19]. Thus, the driving force for the exclusion of the SWNTs from the N* would be the mismatch in the excluded volume (entropic) interactions [12] or elastic inter-particle interactions [20]. Yet, recent studies of hydrophobic particles of different size (from a few nanometers to hundreds of nanometers) and shape (spherical and elongated) indicate that carbonaceous inclusions are excluded from the N*, irrespective of their shape and size. This observation suggests that in the hybrid systems, hydrophobic interactions may play a more significant role than expected [12].

In the study presented here, we investigated the effect of a non-ionic surfactant, BrijS20 (Polyethylene glycol octadecyl ether), and amphiphilic block copolymers, Poly(ethylene oxide)-poly(propylene oxide)-poly(ethyleneoxide), namely F108 and F127 (Pluronics, BASF), on the co-assembly of SWNTs and CNCs in aqueous suspensions at the bi-phasic regime of the CNCs. The surfactant and the polymers are known to adsorb onto SWNTs and CNCs in aqueous media [21,22,23]. While of a similar chemical composition, the molecular assemblies formed by the three surface-active molecules are of different dimensions, enabling us to probe geometrical size-related effects in the hybrid systems comprising CNC–surfactant/polymer–SWNT systems.

SAXS investigation reveals that the adsorbed molecules modify the dimensions of the decorated CNCs, while the characteristic inter-particle distance (*d*_0_) of the liquid crystalline native CNCs phase is preserved. The (block-copolymer) micelles (or chains) do not induce depletion of the CNCs rods. Furthermore, the chiral nature of the N* phase is preserved in the CNC–surfactant–SWNT hybrids, and the pitch is significantly reduced as compared to the native CNC phases at a similar concentration. While the surfactant-decorated SWNT are not depleted from the N* of the CNCs, they are not incorporated into the chiral nematic structure, but rather form an independent network with a mesh-size of the order of hundreds of nanometers. Thus, decoration of the CNCs and the SWNTs by surfactant molecules (or block-copolymer) mediates the inter-particle interactions and enables the assembly of the N* phase of the CNCs within a percolated SWNT network. These findings may be used as guidelines for utilizing non-ionic surfactants and block copolymers for engineering hybrid nanocomposites based on the LC phases of CNCs.

## 2. Materials and Methods

### 2.1. Materials

#### 2.1.1. Cellulose Nanocrystals (CNCs)

CNCs suspension (12 wt%, Na^+^ counterion) was purchased from Cellulose Lab, Fredericton, Canada (CAS# 9004-34-6, https://www.celluloselab.com, accessed 12 November 2021). The reported dimensions of the CNCs are 5–20 nm in width and 140–200 nm in length. In aqueous suspensions, CNC rods are negatively charged due to the half-sulfate ester groups on their surface (pKa~1.99) [24].

#### 2.1.2. Single-Walled Carbon Nanotubes (SWNTs)

SWNTs from two different sources were used as received: SWNTAP (CarboLex Inc., Lexington, KY, USA (http://carbolex.com, accessed 12 November 2021) synthesized by arc discharge) and SWNTCH (CheapTubes, Inc., Grafton, VT, USA (http://www.cheaptubes.com, accessed 12 November 2021). The SWNTs are characterized by a typical diameter of 1.3−1.4 nm and lengths ranging from hundreds of nanometers to tens of microns. Reported impurities consist of graphite, metal catalysts, and amorphous carbon.

#### 2.1.3. Surfactants and Block Copolymers

BrijS20 (Merck, Israel, CAS# 9005-00-9). Poly(ethylene glycol)-*block*-poly(propylene glycol)-*block*-poly(ethylene glycol), PEO_132_PPO_50_PEO_132_ (F108, product no. 583 062) and PEO_100_PPO_70_PEO_100_ (F127, product no. 583 106) were received as a gift from BASF and used as received (Table 1 and Figure S1 of the SI).

### 2.2. Preparation Methods

#### 2.2.1. Preparation of Solutions

BrijS20 aqueous solutions were prepared by stirring the powder in Millipore water (18.2 MΩ cm). Aqueous solutions of F108 and F127 were prepared by vigorous stirring for 24 h at 0 °C and incubation at 4 °C for 3–4 days, for complete dissolution.

#### 2.2.2. Preparation of CNC Suspensions

As-received CNC slurry (12 wt% suspension in water) was tip-sonicated (Ultrasonic processor model VCX-130, Sonics & Materials Inc., Newtown, CT, USA, 130 W, 20 kHz) at 30% amplitude for 4 min. The 12 wt% CNCs suspension was sonicated in two sessions of two minutes. All suspensions were cooled during sonication to prevent the hydrolysis of the sulfate groups at the CNC surface due to overheating [31,32]. The suspensions were diluted to the desired concentration using Millipore water. The volume fraction of the CNC suspension (*φ*) is calculated according to the relation: $\varphi =\varphi_{w}\rho_{s}/\left[\rho_{CNCs}-\varphi_{w}\left(\rho_{CNCs}-\rho_{s}\right)\right]$, where *φ_w_* is the CNC weight fraction (*w*/*w*), *ρ_s_* is the solvent density, and *ρ_CNCs_* is CNC density (1.5 g∙cm^−3^). 

#### 2.2.3. Preparation of Dispersions of SWNTs

Sonication-assisted dispersion of SWNTs was carried out following previously published protocols [33,34]. For details see the SI (Figure S2). The reported concentrations of the SWNTs relate to the initial concentration of the SWNTs in the dispersions. 

### 2.3. Characterization

#### 2.3.1. Electrokinetic Mobility

Mobility measurements were carried out in 0.1 wt% suspensions of CNCs using the Zetasizer Nano ZS (Malvern Instruments Ltd., Almelo, The Netherlands) (Table S1 of the SI). 

#### 2.3.2. Visual Inspection

The volume fraction of the non-isotropic phase *ϕ*_LC_ was determined by visual inspection of the samples.

#### 2.3.3. Polarized Optical Microscopy (POM)

POM images of CNC suspensions were taken using Olympus BX53-F2 microscope equipped with a high-resolution Olympus DP74 camera in crossed polarizer configuration and analyzed using ImageJ. The pitch was calculated from the measured distance between the regularly spaced extinction lines (“fingerprint pattern”) observed due to the helical rotation of the director in the direction orthogonal to the long axis of the CNCs rods in the chiral nematic phase [15,35].

#### 2.3.4. Small-Angle X-ray Scattering (SAXS)

Scattering patterns of CNCs suspensions were collected using SAXSLAB GANESHA 300-XL Xenocs, Grenoble, France. CuΚα radiation was generated by a Genix 3D Cu-source with an integrated monochromator, 3-pinhole collimation, and a two-dimensional Pilatus 300 K detector. The scattering intensity I(q) was recorded in the λ interval of 0.007 < q < 0.25 Å^−1^ (corresponding to the length scale of 25–900 Å), where the scattering vector is defined as q = (4π/λ) sin θ, with 2θ and λ being the scattering angle and wavelength, respectively. The measurements were performed under vacuum at ambient temperature. The suspensions were sealed in thin-walled quartz capillaries about 1.5 mm in diameter and with 0.01 mm wall thickness. The scattering curves were corrected for counting time and sample absorption. The 2D SAXS patterns were azimuthally averaged to produce one-dimensional intensity profiles, I vs. q, using the two-dimensional data reduction program SAXSGUI. The scattering spectra of the solvent were subtracted from the corresponding solution data using Igor Pro 9 from WaveMetrix Portland Oragon for analysis of small-angle scattering data [36]. Data analysis was based on fitting the scattering curve to an appropriate model provided by Mao et al. [37] using Wolfram Mathematica 12.3 software, Champaign, IL, USA. 

#### 2.3.5. Raman Spectroscopy

Raman scattering spectra were obtained using a Horiba LabRam HR micro-Raman system, equipped with a Syncerity CCD detector deep-cooled to −60 °C, 1024 × 256 pixels. The excitation source was a 532 nm laser used at 0.1–1% of the maximal laser power, about 0.5 mW. The laser was focused with a 50 × LWD objective (Olympus LMPlanFL-N, NA = 0.5) to a spot of about 1.3 μm. The measurements were taken with a 600 g mm^−1^ grating and a 100 μm confocal microscope hole. The typical exposure time was 60 milliseconds. 

#### 2.3.6. Transmission Electron Microscopy (TEM) Imaging

Cryo-TEM: rapid cooling enables direct imaging of molecular assemblies and nanostructures in aqueous media. The samples were prepared by applying a 3 μL drop to a TEM grid (300 mesh Cu Lacey substrate, Ted Pella, Ltd., Redding, CA, USA) following a short pre-treatment of the grid via glow discharge. The excess liquid was blotted, and the specimen was vitrified by rapid plunging into liquid ethane precooled by liquid nitrogen using a vitrification robot system (Vitrobot mark IV, FEI). The rapid cooling results in physical fixation of the liquid state so as to preserve the native structures. Thus, it allows examination of the polymeric assemblies in the high vacuum of the electron microscope at cryogenic temperature, which prevents the formation of either cubic or hexagonal ice. The vitrified samples were examined at −177 °C using FEI Tecnai 12 G^2^ TWIN TEM (FEI, Hillsboro, OR, USA) operated at 120 kV and equipped with a Gatan model 626 cold stage. The images were recorded by a 4 K × 4 K FEI Eagle CCD camera in low dose mode. TIA (Tecnai Imaging and Analysis, Tecnai 3.0 FEI, Hillsboro, OR, USA) was used to record the images.

Negative staining: to increase the inherently low contrast of the polymer assemblies, transmission electron microscopy of dried, stained samples was performed. The solution (2 μL) was applied to a glow-discharged TEM grid (carbon supported film on 300 mesh Cu grids, Ted Pella, Ltd., Redding, CA, USA). The excess liquid was blotted, and the grids were washed on two droplets of de-ionized water following by staining with 2% uranyl acetate for 40 s. The grids were blotted and dried under ambient conditions at room temperature before they were observed by FEI Tecnai 12 G^2^ TWIN TEM operated at 120 kV. The images were recorded by a 4 K × 4 K FEI Eagle CCD camera using TIA (Tecnai Imaging and Analysis, Tecnai 3.0 FEI, Hillsboro, OR, USA).

## 3. Results

### 3.1. SWNTs Dispersions

Sonication-assisted dispersions of SWNTs (0.1 wt%) were prepared in aqueous solutions of BrijS20 (1 wt%), F108 (2 wt%), and F127 (2 wt%) following previously published protocols [26]. The surfactants were used at the minimal concentrations needed for preparation of stable dispersions of individual SWNTs [21,26,33]. As polymer adsorption is non-reversible [21], post-dispersion mixing with the CNC suspension did not affect the stability of the dispersion. Note that F108 was used at a concentration below the CMC of the polymer. Cryo-TEM images of the dispersions indicate that the SWNTs are dispersed as individual, non-bundled tubes (Figure S2 of the SI).

### 3.2. CNs–Surfactant/Polymer Suspensions

CNC suspensions were mixed with aqueous solutions of BrijS20, F108 and F127 to final concentrations of 6 wt% CNCs and 0.5 wt% BrijS20, 1 wt% F127 or F108.

**Figure 1:**
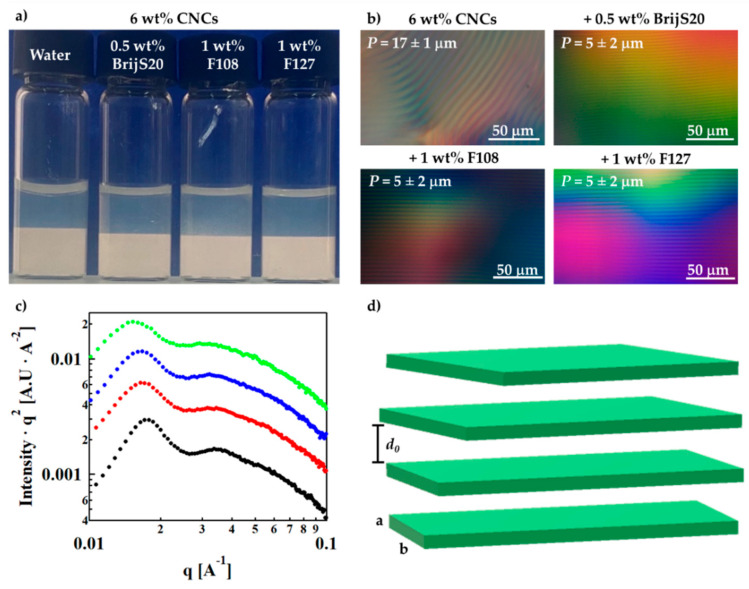
Suspensions of 6 wt% CNCs. (**a**) Image of vials taken between crossed polarizers, (**b**) POM images of the (lower) N* phase, (**c**) Lorentz-corrected intensity curves of 6 wt% CNCs: in water (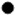), in BrijS20 solution (0.5 wt%) (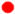), in F108 solution (1 wt%) (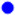) and in F127 solution (1 wt%) (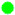). The curves are shifted for better visualization. SAXS I vs. q curves are presented in Figure S5 of the SI (**d**) Schematic illustration of the parallelepiped stacking model by Mao et al. [37].

Optical images taken between crossed polarizers (Figure 1a) show an isotropic upper phase and a lower birefringent phase. The volume fraction of the two phases is similar in CNC suspensions in water (left) and CNCs in surfactant/polymer solutions.

Zeta potential measurements of CNCs indicate that the surface potential of the CNCs in aqueous suspensions is −52 ± 2 mV. The value is reduced in the presence of the surface-active molecules, indicating adsorption (Table 2).

POM images show an iridescent lower phase (Figure 1b) with a typical pitch of *P* = 5 ± 2 μm in the CNC–surfactant/polymer suspensions as compared to *P* = 17 ± 1 μm in the CNC–surfactant/polymer suspensions as compared to *P* = 17 ± 1 μm in the surfactant-free CNCs suspensions. The reduction in the pitch in the presence of the surfactant/polymer at a given CNC concentration (here, 6 wt%) indicates a higher rotation of the director and was reported before for polymer-decorated CNCs [38].

Cryo-TEM images of the CNC water suspensions and CNC–BrijS20 mixture presented in Figures S3 and S4 of the SI show that the two phases retain their typical inter-particle distance and the orientational order of the CNCs.

The nanostructure of the CNC–surfactant/polymer suspensions was investigated using SAXS. In Figure 1c, we present the Lorentz-corrected curves of CNC suspensions in polymer and surfactant solutions (I vs. q SAXS curves are presented in Figure S5 of the SI). The curves exhibit a first-order peak, indicative of a typical distance between suspended CNCs *d*_0_ = 2π/q_0_, and a shoulder of a second-order interference peak (q_1_), at values corresponding to a ratio of 2:1 (Table S2 of the SI), characteristic of the native lamellar periodic structures of the CNCs mesophase at this concentration [39]. The initial slope in the low q regime of the scattering curves is somewhat reduced (I vs. q curves, Figure S5 of the SI), indicating a modification of the structure factor due to some screening of the interparticle interactions.

The SAXS curves (Figure S5 of the SI) were fitted to the parallelepiped stacking model developed by Mao et al. [37,40] where an individual CNC particle is modeled by a parallelepiped with a length *L*, width *b*, and thickness *a* stacked in one direction with a distance *d*_0_ between the surfaces of two adjacent particles (Figure 1d). The fitting parameters presented in Table 3 indicate that the typical interparticle distance *d*_0_ is similar in the different suspensions. The typical thickness (Figure 1d), *a*, is increased by about 15%, while the most significant effect is on the width, *b*: the width is doubled for most of the additives and is slightly higher in the F127 suspensions than in the BrijS20 suspensions (Table 3). The latter may indicate increased aggregation of the CNCs in the presence of the surfactants, probably due to reduced surface charge [41].

The SAXS results, along with the reduction in the zeta potential are consistent with adsorption of the surface-active molecules onto the CNCs particles.

### 3.3. CNC–Surfactant/Polymer–SWNTs Dispersions

**Figure 2:**
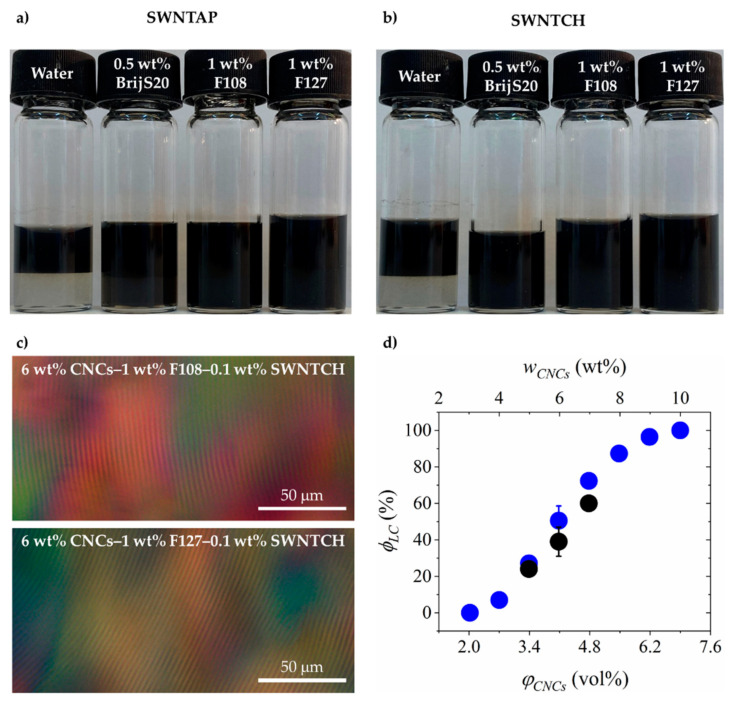
Optical images of CNC–surfactant/polymer–SWNT mixtures: (**a**) SWNTAP and (**b**) SWNTCH. In each image, from left to right: surfactant-free dispersion of SWNTs in CNCs, CNC–BrijS20–SWNTs, CNC–F108–SWNTs, and CNC–F127–SWNTs. (**c**) POM images of CNC–F108–SWNTs and CNC–F127–SWNTs. (**d**) Volume fraction of the N* phase (*ϕ_LC_*) as a function of CNC concentration in surfactant-free (water) CNC suspensions (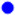) and in CNC–BrijS20–SWNTCH dispersions (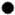).

Liquid mixtures of CNC–surfactant/polymer–SWNTs were prepared as described above. The images (Figure 2a,b) clearly show that while surfactant-free, CNC-dispersed SWNTs are depleted from the lower phase of the native suspension (left vial in both images) they are dispersed in both the isotropic and N* phase of the CNC–surfactant/polymer suspensions. In these mixtures the chiral nematic structure is preserved as demonstrated in representative POM images of the hybrid CNC–F108–SWNTs and CNC–F127–SWNTs (Figure 2c and Figure S8 of the SI) with a pitch value of 5 ± 2 μm, as well as the volume fraction of the N* phase (Figure 2d). Thus, the surfactant-decorated SWNTs are neither depleted from the N*, nor do they modify the pitch or the macroscopic phase diagram of the surfactant/polymer-decorated CNCs. The observation was reproducible for the two different types of SWNTs.

**Figure 3:**
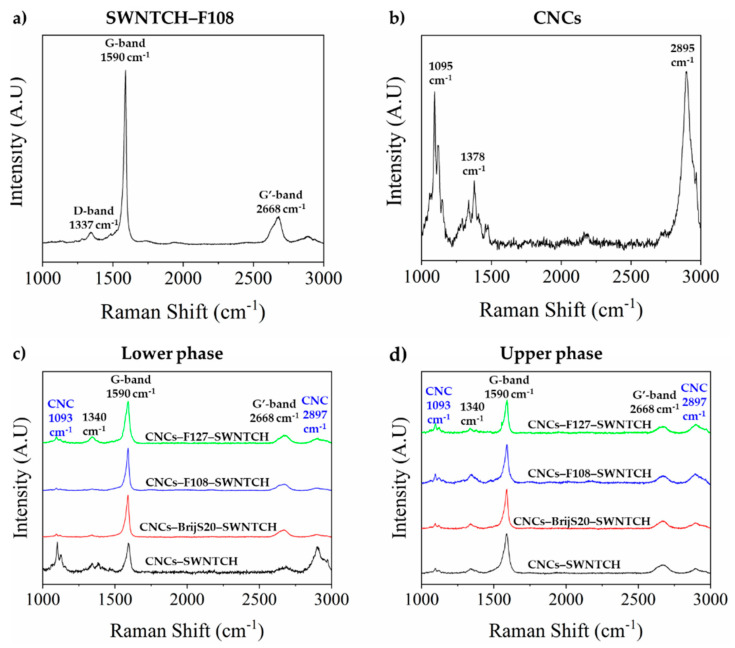
Raman scattering of (**a**) F108–SWNTCH dried dispersion, (**b**) CNC powder, and (**c**) lower and (**d**) upper phases of dried films prepared from dispersions of: CNC–SWNTCH (**—**), CNC–BrijS20–SWNTCH (**—**), CNC–F108–SWNTCH (**—**) and CNC–F127–SWNTCH (**—**).

Raman spectroscopy was used to determine the SWNT-to-CNC ratio in the two phases. The Raman spectra of the pristine materials are presented in Figure 3a,b. For SWNTCH, a tangential stretching mode (G-band) is observed at ~1590 cm^−1^ and a broader band around ~2670 cm^−1^ (G′-band) arises from an overtone of the disorder induced mode around 1340 cm^−1^ (D-band) [42]. For CNCs, two sharp peaks are observed at ~1095 cm^−^^1^ and ~2895 cm^−^^1^_,_ characteristic of the C-O ring and C-H stretching, respectively. The broader band observed at ~1380 cm^−1^ is attributed to HCC, HCO and HOC bending [43,44,45]. The ratio between the D-band and the G-band intensities is similar to that of the pristine SWNTs [46]. The latter indicates that the interaction between the SWNTs and the adsorbed surfactants does not introduce additional defects and does not modify the sp^2^ hybridization of the pristine SWNTs.

The relative fraction of SWNTs is calculated from the ratio of the intensities of the SWNTs G-band at ~1590 cm^−1^ to CNCs characteristic peak at ~1093 cm^−1^.

The Raman spectra of samples dried from the *I* phase (upper, isotropic) and the N* phase (lower, chiral nematic) of CNC–surfactant/polymer–SWNTCH dispersions are presented in Figure 3c,d.

In CNC–SWNT suspensions, in the absence of surfactants (black curve in Figure 3c,d), the SWNTs are depleted from the N* phase and the SWNTs enrich the isotropic (upper) phase to a ratio of 8:1 (I/N*) (and 5:1 (I/N*) for SWNTAP, see Figures S6 and S7 of the SI).

Very differently, in the CNC–surfactant/polymer–SWNTs dispersions, the SWNTs are either equally distributed between the two phases (I/N* 1:1 ratio) or enriched in the N* phase (Table S3 of the SI).

**Figure 4:**
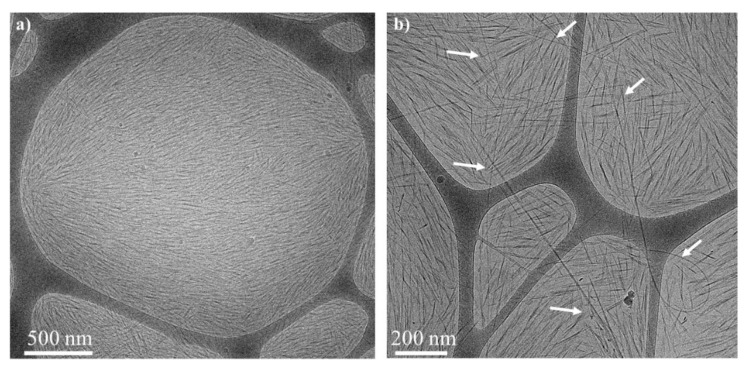
Cryo-TEM images of the N* phase of CNC–BrijS20–SWNTCH dispersions (**a**), (**b**) at two different magnifications. The arrows in (**b**) point to the contour line of the SWNTCH.

**Figure 5:**
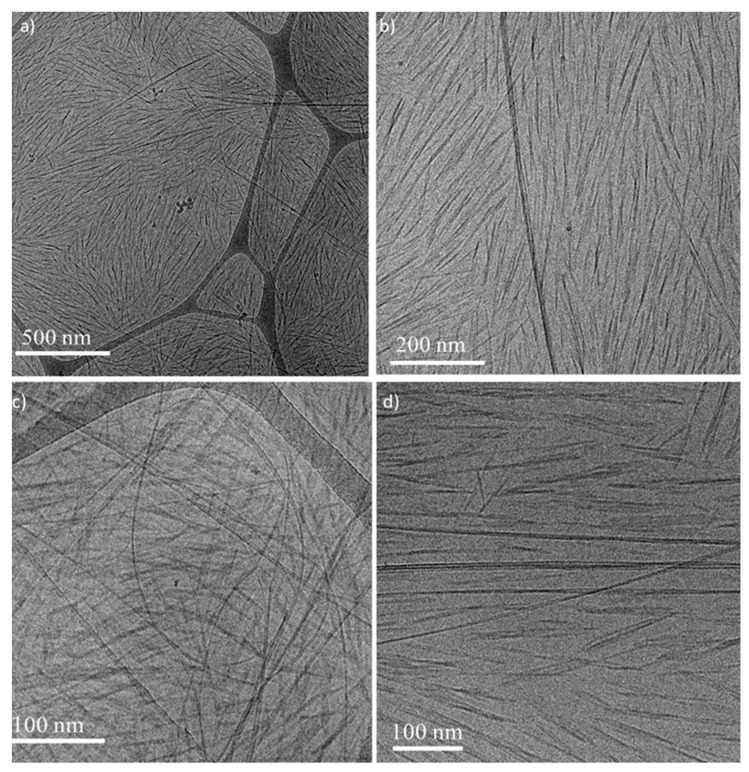
Cryo-TEM images of (**a**,**b**) the N* phase of CNC–F108–SWNTCH suspensions. (**c**) The upper (isotropic) and (**d**) lower (N*) phase of CNC–F127–SWNTCH.

Cryo-TEM images of the N* phase of a CNC–BrijS20–SWNTCH mixtures are presented in Figure 4. As in the native suspensions, the CNC rods are oriented over hundreds of nanometers. The SWNTs cannot be resolved at this magnification (Figure 4a), but at a higher magnification (Figure 4b) a fraction of the SWNTs network with typical mesh size ranging from hundreds of nanometers to microns is observed. At even higher magnifications (Figure S4 of the SI), Brij micelles can be observed. Similar structures are observed in cryo-TEM images of CNC–F108–SWNTs (Figure 5a,b) and CNC–F127–SWNTs (Figure 5c,d). Note that the Pluronic micelles (or chains in the case of F108) are not observed in cryo-TEM due to the low contrast of PEO with water [47].

**Figure 6:**
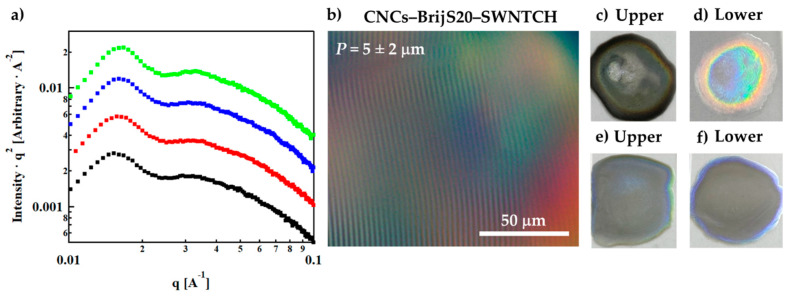
(**a**) SAXS Lorentz-corrected intensities of the lower phase of CNCs–1 wt% F127 (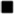), 6 wt% CNCs–0.5 wt% BrijS20–0.1 wt% SWNTCH (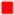), 6 wt% CNCs–1 wt% F108–0.1 wt% SWNTCH (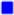) and 6 wt% CNCs–1 wt% F127–0.1 wt% SWNTCH (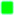). The curves are shifted for better visualization. (**b**) POM image of the N* phase of 6 wt% CNCs–0.5 wt% BrijS20–0.1 wt% SWNTCH mixture. Dried films of the (**c**) upper and (**d**) lower phases of 6 wt% CNCs–0.1 wt% SWNTCH, and (**e**) upper and (**f**) lower phases of 6 wt% CNCs–0.5 wt% BrijS20–0.1 wt% SWNTCH.

The Lorentz-corrected SAXS curves obtained from CNC–surfactant/polymer–SWNT dispersions are presented in Figure 6a. Fitting of the 1D scattering curves to the stacking model (Table 3 and Table S4; Figure S9 of the SI where scattering curves of CNC–surfactants and CNC–surfactant–SWNTs are presented) reveals that the presence of the SWNTs does not affect the lateral dimensions *(a*, *b)* of a surfactant/polymer-decorated CNC rod nor the interparticle distance *d*_0_. Furthermore, the POM image (Figure 6b), indicates that the chiral nature (N*) is preserved and the typical pitch does not change, as compared to the pitch measured in the absence of SWNTs, for CNC–surfactant/polymer suspensions (~5 μm).

Optical images of thin films dried from surfactant-free CNC suspensions of SWNTCH, and surfactant-mediated CNC–BrijS20–SWNTCH suspensions are presented in Figure 6c–f. While the film formed by drying of the optically isotropic (upper) phase of the surfactant-free CNC–SWNTCH suspension is black (Figure 6c), the film dried from the lower phase is bright (Figure 6d), indicating that the SWNTs are depleted from this phase. The dark rim of the dried (*I*) phase (Figure 6c) indicates segregation of dispersed SWNTs to the perimeter of the drop. In contrast, films prepared in a similar way from the *I* and N* of CNC–BrijS20–SWNTCH suspensions (Figure 6e–f) are both homogenously dark, indicating that the SWNTs are equally distributed in the two phases. In these films (Figure 6e–f) the rims are somewhat brighter than the inner part of the film. 

## 4. Discussion

The coexistence of surfactant (or polymer)-decorated SWNT networks and surfactant (or polymer)-decorated CNC LC mesophases in the presence of non-ionic surfactants (or triblock copolymers) was observed. Raman measurements indicate that the SWNTs are equally distributed between the two phases and are not excluded from the N* phase of the CNCs. 

**Figure 7:**
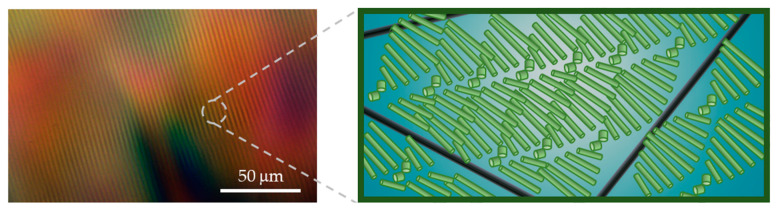
CNCs–1% F108–SWNTCH. POM image (**left**) and schematic illustration (not to scale) of the two co-existing networks (**right**): chiral mesostructure of CNCs (light green) and percolated SWNT network (black). The surfactants decorating the nanostructures and their assemblies in solution are not shown in the sketch.

Cryo-TEM images clearly show percolated networks of individual SWNTs in both phases. The mesh-size of the SWNTs network is of the order of hundreds of nanometers to microns, and the SWNTs are not incorporated into the chiral nematic structure but rather form an independent network (Figure 7).

Molecular CNC–surfactant/polymer–SWNT assemblies of different typical dimensions are formed in solutions of BrijS20 micelles (radius~3 nm), individual chains of F108 (hydrodynamic radius~4 nm), and F127 core–shell micelles with a radius of about 13 nm [25,26,27,28,29,30]. Yet, the observed behavior of the three-component (CNC–surfactant/polymer–SWNT) hybrid system is similar. At this point, it is not possible to deduce whether individual molecules, micelles, or hemi-micelles decorate the CNCs. Clearly, the nanostructure of the chiral nematic phase is hardly altered by the presence of the surfactants or the SWNTs networks and size-selective exclusion of micelle (polymer)-decorated SWNTs is not observed.

The key to CNC–SWNT coexistence is probably the presence of non-ionic surfactants. These are known to act as mediating agents in mixtures of nanostructures enabling the preparation of nano-hybrids, and were shown to improve the homogeneity of dried CNC films [48]. It is observed here that the suspensions can be dried, leading to the formation of thin-film hybrids with macroscopically homogeneous distribution of the SWNTs, without segregation of the SWNTs to the perimeter of the dried films.

Detailed analysis of the CNC–surfactant/polymer–SWNT mixtures indicates that the macroscopic phase diagram and inter-particle distance (*d*_0_) characteristic of the N* phase of the native CNCs suspensions, as measured via SAXS, are preserved in the rod–sphere mixtures, while the effective thickness of the surfactant-decorated CNCs is increased. These observations suggest that the Brij20 and F127 micelles and the F108 chains (at the low concentrations investigated here) do not induce depletion [49] of the CNC rods. Depletion interactions between the micelles and the CNCs would have led to crowding of the CNCs and a reduction in the inter-CNC distance.

POM imaging of CNC–surfactant and the CNC–surfactant–SWNT phases clearly shows the typical fingerprint pattern characteristic of the N* phase of the CNCs. The origins of chirality of the LC CNC phase may be geometrical or related to charge distribution at the surface [7,50]. Thus, one would expect that adsorption of micelles or polymer molecules would screen the chiral interactions. Yet, here we observe that the pitch of the N* phase is reduced (*P*~5 μm) as compared to the native phase (*P*~17 μm). Shortening of the helix of the N* phase at a constant CNC volume fraction, indicates that the rotation of the director is higher, probably due to modification of the inter-CNC interactions among the rods [7] and strengthening the chiral interactions. The pitch is not further affected by the presence of the SWNTs. We note here that helical pitch (an equilibrium property of the N* phase) is not associated with *d*_0_ (inter-particle distance).

## 5. Conclusions

Liquid suspensions of CNCs and SWNTs can be used as colloidal inks for additive manufacturing [51] and layer-by-layer deposition [18] of multifunctional nanocomposites with novel combinations of optical, electrical, mechanical, and thermal properties [18,52,53,54]. Yet, mismatch in the physical characteristics of the two components, including diameter, aspect ratio, rigidity, and interfacial interactions, results in segregation of the components and depletion of the SWNTs from the liquid–crystalline phases of the CNCs [12]. 

In this study, we report the utilization of non-ionic, surface-active molecules for the preparation of hybrid liquid phases comprising CNC and SWNT networks. It was observed that the surfactant-decorated SWNTs are neither excluded from the surfactant-decorated CNC phases, nor are they incorporated into the nematic (N*) structure.

Rather, cryo-TEM imaging reveals that the SWNTs networks co-exist with the surfactant-mediated phases of the CNCs, and Raman spectroscopy indicates that the SWNTs distribute equally between the isotropic and chiral nematic phases of the CNCs. Detailed SAXS and cryo-TEM characterization of the emerging phases show that adsorbed surfactants do not disturb the nematic structure of the CNCs mesophase and POM imaging indicates that the chiral nature of the N* phase is preserved, while the pitch is reduced.

These findings indicate that non-ionic surfactants can be used for engineering of the interfacial interactions in hybrid mixtures of CNCs and SWNTs. The resulting mixtures could be used for liquid processing and deposition of multi-component CNC-based functional nanocomposites.

## Figures and Tables

**Table 1:** Properties of BrijS20 and Pluronics triblock copolymers [25,26,27,28,29,30].

Surfactant/Polymer	Mn	CMC (25 °C) (wt%)	Diameter of the Micelles (25 °C) (nm)
BrijS20	1152	7.6 × 10^−4^	5–8
F108	14,600	4.5 *	15–20
F127	12,500	0.7	20–25

* F108 was used at concentrations below CMC. The hydrodynamic diameter of F108 is about 8 nm (25 °C).

**Table 2:** Calculated zeta potential in 0.1 wt% CNCs.

Surfactant/Polymer (wt%)	Zeta Potential (mV)		
	BrijS20	F108	F127
0.1	−36 ± 1	−44 ± 3	−44 ± 3
0.2	−29 ± 2	−43± 1	−44 ± 1

The measured mobility presented in Table S1 of the SI.

**Table 3:** The typical thickness 〈a〉, width 〈b〉, and the inter-plate distance $\langle d_{0}\rangle$ as determined from the parallelepiped stacking model.

Sample	〈a〉(nm)	〈b〉(nm)	$\langle d_{0}\rangle (\mathrm{nm})$
6 wt% CNCs	2.6	20	35 ± 10
6 wt% CNCs–0.5 wt% BrijS20	3.1	39	36 ± 11
6 wt% CNCs–1 wt% F108	3.3	44	37 ± 12
6 wt% CNCs–1% F127	3.1	46	40 ± 13
6 wt% CNC–0.5 wt% BrijS20–0.1 wt% SWNTCH	3.0	41	38 ± 12
6 wt% CNCs–1 wt% F108–0.1 wt% SWNTCH	3.2	42	38 ± 12
6 wt% CNCs–1 wt% F127–0.1 wt% SWNTCH	3.2	45	37 ± 12

## Data Availability

The data presented in this study are available on request from the corresponding author.

## Supplementary Materials

The following are available online at https://www.mdpi.com/article/10.3390/nano11113059/s1, Figures S1–S4 TEM images, Table S1 electrokinetic data, Figures S5 and S9, Tables S2, S4 and S5 SAXS scattering curves and data, Figures S6, S7 and Table S3 Raman spectra and data, Figure S8 POM images.
