# Supplementary material for: Surfactant-Mediated Co-Existence of Single-Walled Carbon Nanotube Networks and Cellulose Nanocrystal Mesophases
Source: Nanomaterials (Basel). 2021 Nov 13;11(11):3059. doi: 10.3390/nano11113059 (PMC8624387; doi:10.3390/nano11113059)
Supplement: Supplementary file 1 [file nanomaterials-11-03059-s001.zip › nanomaterials-1428741-supplementary.pdf]

Electronic Supplementary Information for Nanomaterials

Surfactant-mediated co-existence Single-Walled Carbon Nanotubes Networks and Cellulose Nanocrystals mesophases

David Attia[1], Evgenee Yekymov[1], Yulia Shmidov[1], Orit Mendelson[2], Yael Levi-Kalisman[3], Ronit Bitton[1,4] and Rachel Yerushalmi – Rozen [1,4] *

1. Department of Chemical Engineering, Ben-Gurion University of the Negev, 84105 Beer-Sheva, Israel.
2. Department of Chemistry, Nuclear Research Center-Negev, Beer-Sheva 84190, Israel.
3. The Center for Nanoscience and Nanotechnology, and The Institute of Life Sciences, The Hebrew University of Jerusalem, Jerusalem 9190401, Israel
4. The Ilse Katz Institute for Nanoscience and Technology, Ben-Gurion University of the Negev, 84105 Beer-Sheva, Israel.

* Corresponding author: rachely@bgu.ac.il

S1. Micelles dimensions as determined by Negative stain TEM and Cryo-TEM

In Figure S1 we present images of dried, stained samples obtained from micellar solutions. The dark background results from the presence of the heavy Uranyl atoms. The typical radius of BrijS20 micelles of about 5-8 nm (Figure S1a) obtained from TEM measurements (measured over 50 particles) is in agreement with previous reports [1]. The bright spheres observed in the image of the F127 micelles (Figure S1b) show the PPO core of the micelles (diameter of 5-6 nm), while the highly hydrated and much less densely packed PEO shell (where the uranyl ions are not excluded) is not observed [2,3]. SAXS and Small Angle Neutron scattering data reported in the literature were used for determining the micelle's dimensions reported in Table 1 of the main text.

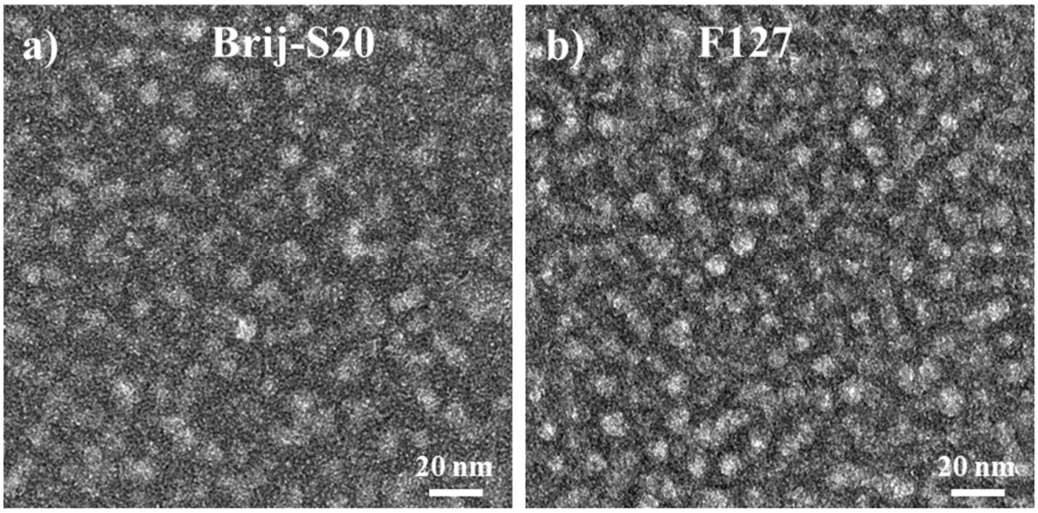

Figure S1. TEM images of negatively stained micellar solutions **(a)** 1 wt% BrijS20 and **(b) 3** wt% F127.

S2. *Dispersions of SWNTs*

Figure S2 presents cryo-TEM images of individual, well-dispersed non-bundled SWNTs (SWNTCH) in aqueous solutions of BrijS20, F108 and F127. The cryo-TEM images show random networks of mostly individual SWNTs formed in the dispersion. At this magnification, and without staining the surfactant and polymer micelles can be hardly observed (but rather contribute to the somewhat grainy background).

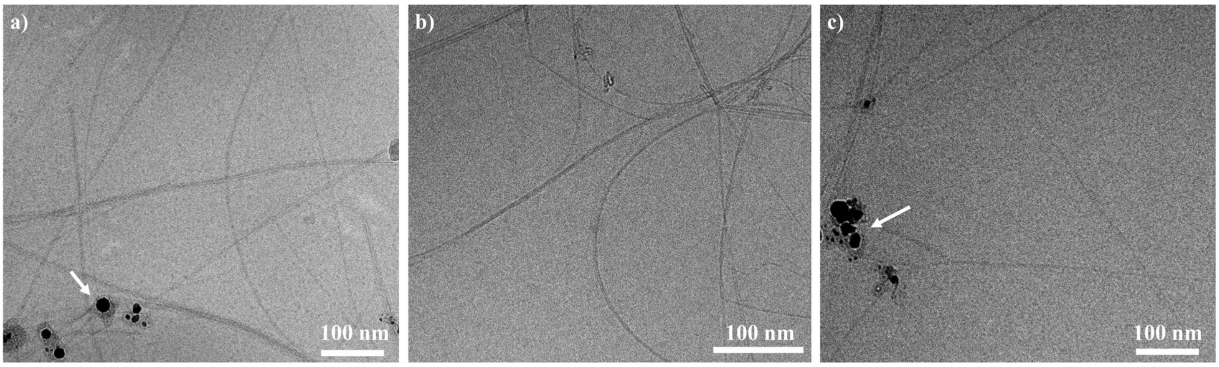

Figure S2. Cryo-TEM images of SWNTs dispersions in **(a)** 1 wt% BrijS20, **(b)** 1 wt% F108 and **(c)** 2 wt% F127. The arrow points at carbon-coated catalyst particles.

S3. *CNCs–surfactant/polymer suspensions*

Table S1 shows the mobility measurements of CNCs–surfactant/polymer aqueous suspensions were carried out using the Zetasizer Nano ZS (Malvern Instruments Ltd.).

Table S1. The Mobility measurements in 0.1 wt% CNCs.

Surfactant/polymer	*Mobility (μmcm $V^{-1}s^{-1}$)*		
(wt%)	BrijS20	F108	F127
0.1	-2.8 ± 0.1	-3.5 ± 0.2	-3.5 ± 0.2
0.2	-2.2 ± 0.1	-3.4 ± 0.1	-3.5 ± 0.1

In Figures S3 and S4 we present cryo-TEM images of the upper (*I*) and lower (*N**) phases of native 6 wt% CNCs suspensions and of 6 wt% CNCs–1 wt% BrijS20, respectively. In Figure S4 the BrijS20 micelles can be observed as well (small dark dots decorating the CNCs and the background).

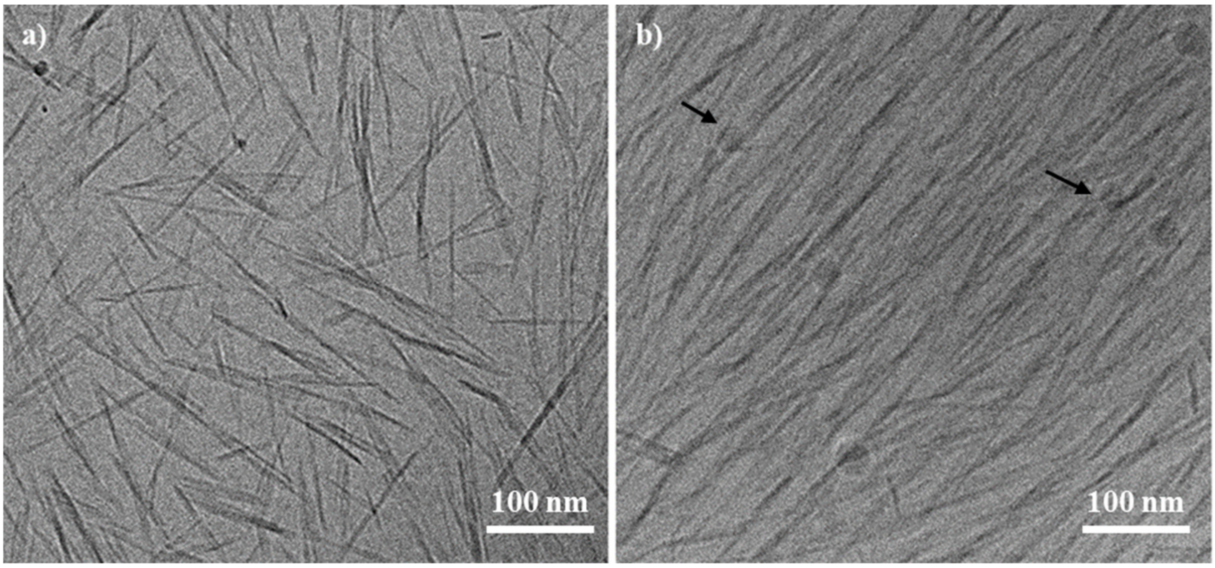

Figure S3. Suspensions of CNCs (6 wt%). **(a)** Upper (*I*) phase **(b)** Lower (*N**) phase. The arrows point at some ice contamination.

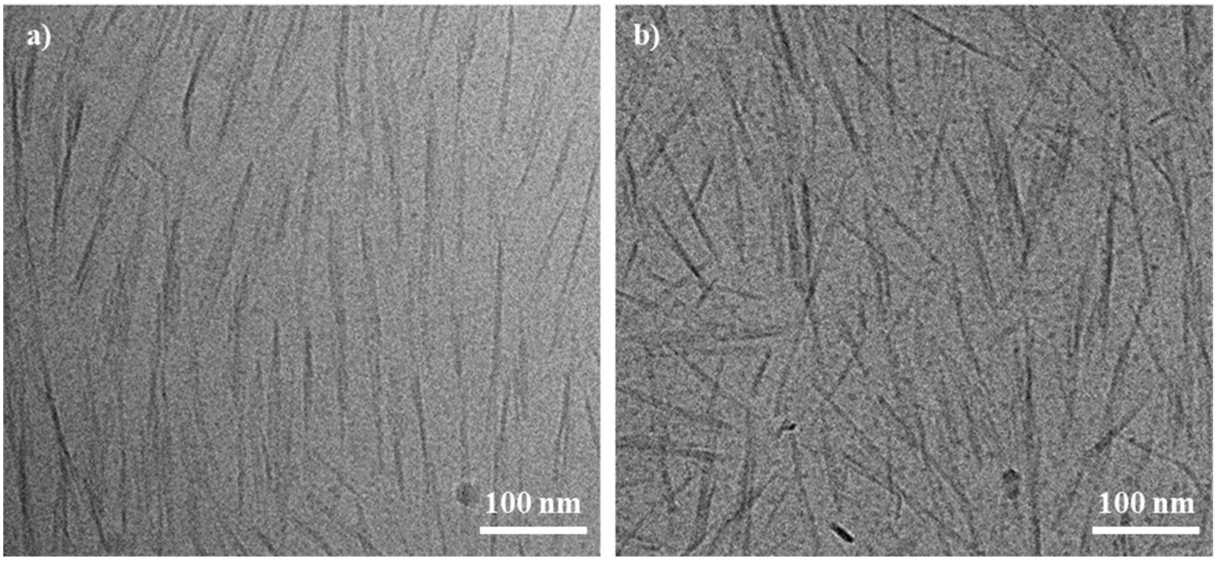

Figure S4. 6 wt% CNCs–1 wt% BrijS20 suspension **(a)** lower (*I*) **(b)** upper (*N**) phase.

Figure S5 exhibits the 1D scattering curves obtained from native 6 wt% CNCs suspension and CNCs-surfactant (or polymer) suspensions. The scattering patterns were fitted to the stacking model developed

by Mao et al. [4,5]. In this model, an individual CNC particle is considered as a parallelepiped with a length L, width b, and thickness a, where the rectangular shaped cross section represents the unit cell dimensions of cellulose crystals, and the CNC particles are stacked in one direction with a distance d_0 between the surfaces of two adjacent particles. The best fit to the parallelepiped model is presented in Figure S5. The parameters that can be obtained from the parallelepiped stacking model fitting are presented in the main text in Table 3. Table S1 presents the volume fraction of the nonisotropic phase ϕ_{LC} (visual inspection), pitch P (measured by POM) and data obtained from SAXS measurements.

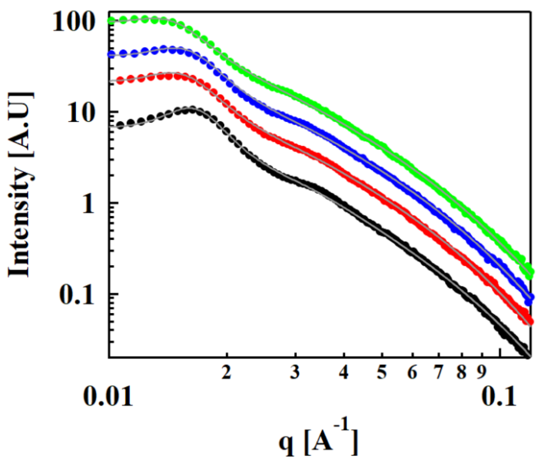

Figure S5. SAXS curves of CNCs–surfactant/polymer suspensions: Native 6 wt% CNCs (●) 6 wt% CNCs–0.5 wt% BrijS20 (●), 6 wt% CNCs–1% F108 (●) and 6 wt% CNCs–1 wt% F127 (●). The solid black lines represent the fit to the stacking model. The curves are shifted for better visualization.

Table S2. The volume fraction of the nonisotropic phase ϕ_{LC}, pitch P, and data from SAXS measurements obtained from the different 6 wt% CNCs–surfactant/polymer suspensions.

Surfactant/polymer	ϕ_{LC} (vol%)	P (µm)	*SAXS*		
			q_0 (A^{-1})	q_1 (A^{-1})	q_1/q_0
Native 6 wt% CNCs	0.5	17 ± 1	0.018	0.035	2:1
0.5 wt% Brij-S20	0.5	5 ± 2	0.017	0.034	2:1
1 wt% F108	0.5	5 ± 2	0.016	0.031	2:1
1 wt% F127	0.5	5 ± 2	0.016	0.032	2:1

S5. *Raman scattering from CNCs–surfactant/polymer–SWNTs films*

Dried films were prepared by drying the isotropic (upper) and the chiral nematic (lower) phases of the different samples on glass slide under ambient conditions. The Raman spectra of CNCs–surfactant/polymer–SWNTCH and CNCs–surfactant/polymer–SWNTAP are presented in Figures S6 and S7, respectively. Table S3 presents the intensity ratios of the signals (I_G/I_{CNC}).

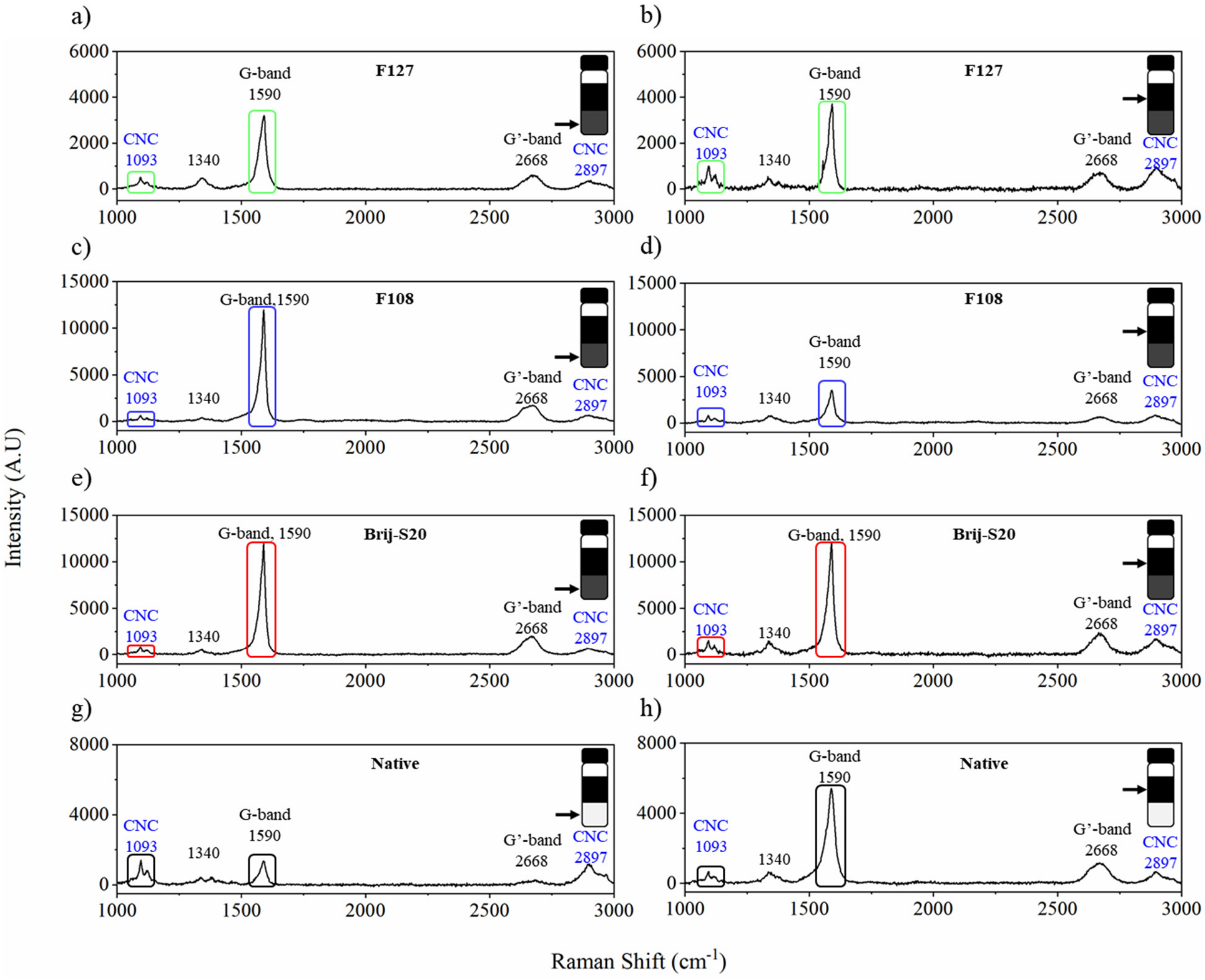

Figure S6. Raman spectra of the lower (a, c, e, g) and upper (b, d, f, h) phases of CNCs–SWNTCH, CNCs–BrijS20–SWNTCH, CNCs–F108–SWNTCH and CNCs–F127–SWNTCH dispersions.

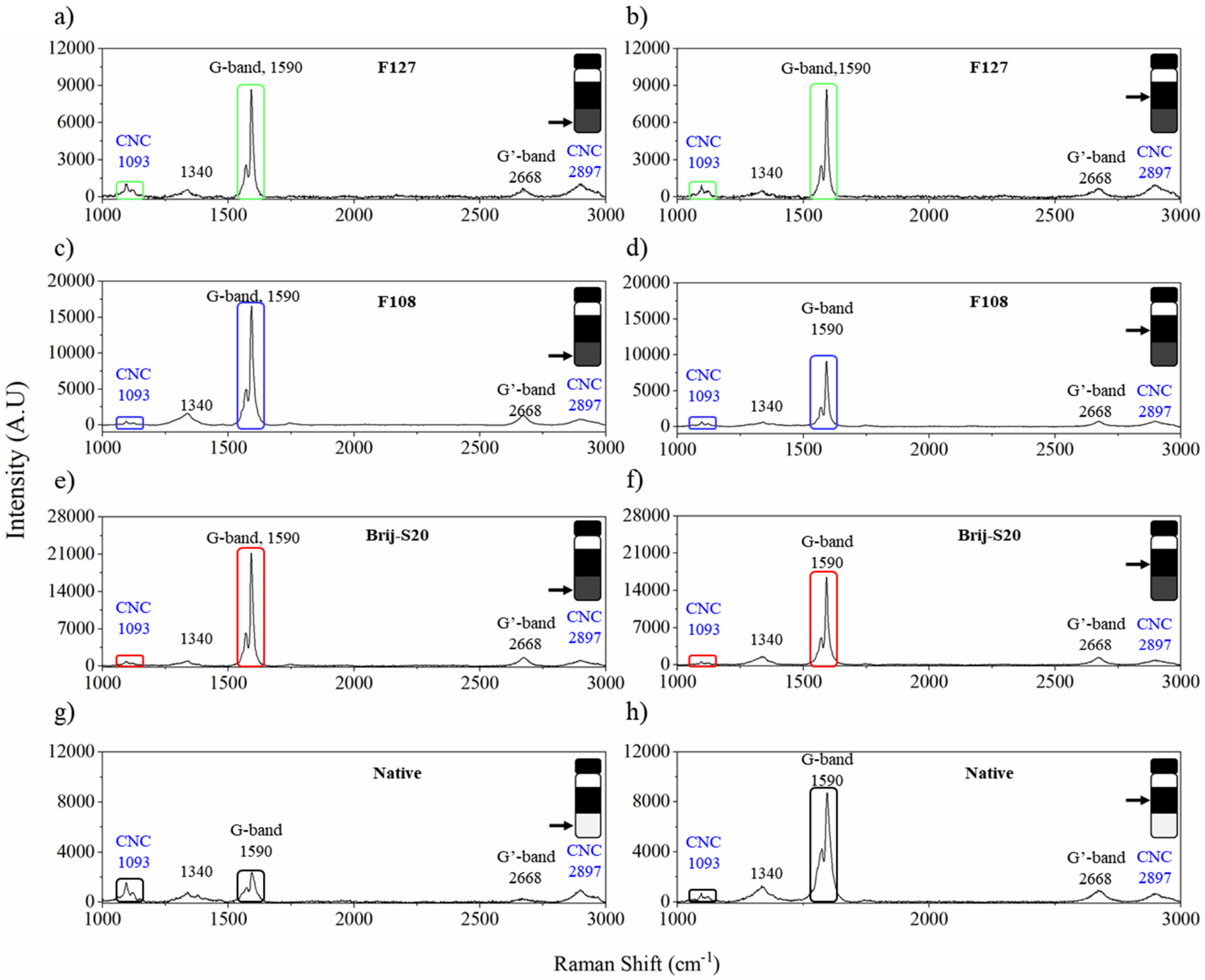

Figure S7. Raman spectra of the lower (a, c, e, g) and upper (b, d, f, h) phases of CNCs–SWNTAP, CNCs–BrijS20–SWNTAP, CNCs–F108–SWNTAP and CNCs–F127–SWNTAP dispersions.

Table S3. The SWNTs /CNCs ratio as evaluated from the ratio of the intensity of the signals (SWNTs G-band at ~ 1590 cm^{-1} to CNC characteristic peak at ~ 1093 cm^{-1}) at the lower and upper phase.

SWNTs	Surfactant/polymer	[SWNTs]/[CNCs] upper phase	[SWNTs]/[CNCs] lower phase	SWNTs(I)/SWNTs(N*) ratio
SWNTCH	6 wt% CNCs	5	1	5:1
	0.5 wt% BrijS20	23	24	1:1
	1 wt% F108	4	18	1:4.5
	1 wt% F127	8	8	1:1
SWNTAP	6 wt% CNCs	8	1	8:1
	0.5 wt% BrijS20	8	13	1:1.6
	1 wt% F108	14	36	1:2.6
	1 wt% F127	3	8	1:1.7

S6. POM images of CNCs–surfactant/polymer–SWNTs mixtures

Figure S8 exhibits the POM images of the tree different hybrid mixtures: CNCs–BrijS20–SWNTs, CNCs–F108–SWNTs and CNCs–F127–SWNTs.

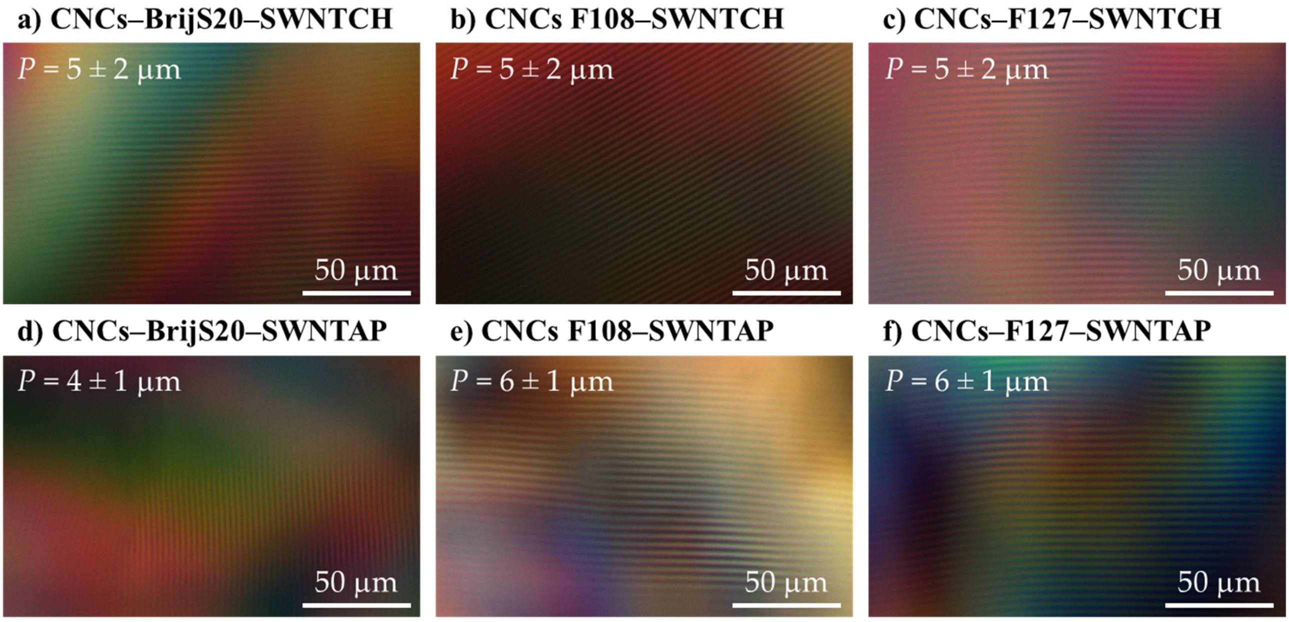

Figure S8. POM images of **(a)** CNCs–BrijS20–SWNTCH, (b) CNCs–F108–SWNTCH, (c) CNCs–F127–SWNTCH, (d) CNCs–BrijS20–SWNTAP, (e) CNCs–F108–SWNTAP and (f) CNCs–F127–SWNTAP.

S7. ***SAXS from CNCs–surfactant/polymer–SWNTs mixtures***

Figure S9 shows SAXS curves obtained from suspensions that contain SWNTs, CNCs and surfactant (or polymer). Here as well, the scattering curves were fitted to the stacking model. Table S4 presents the parameters used for fitting the parallelepiped stacking model for CNCs-surfactant/polymer-SWNTAP. Table S5 exhibit the volume fraction of the nonisotropic phase (visual inspection), pitch (measured by POM) and interparticle distance of the different suspensions (calculated using the stacking model).

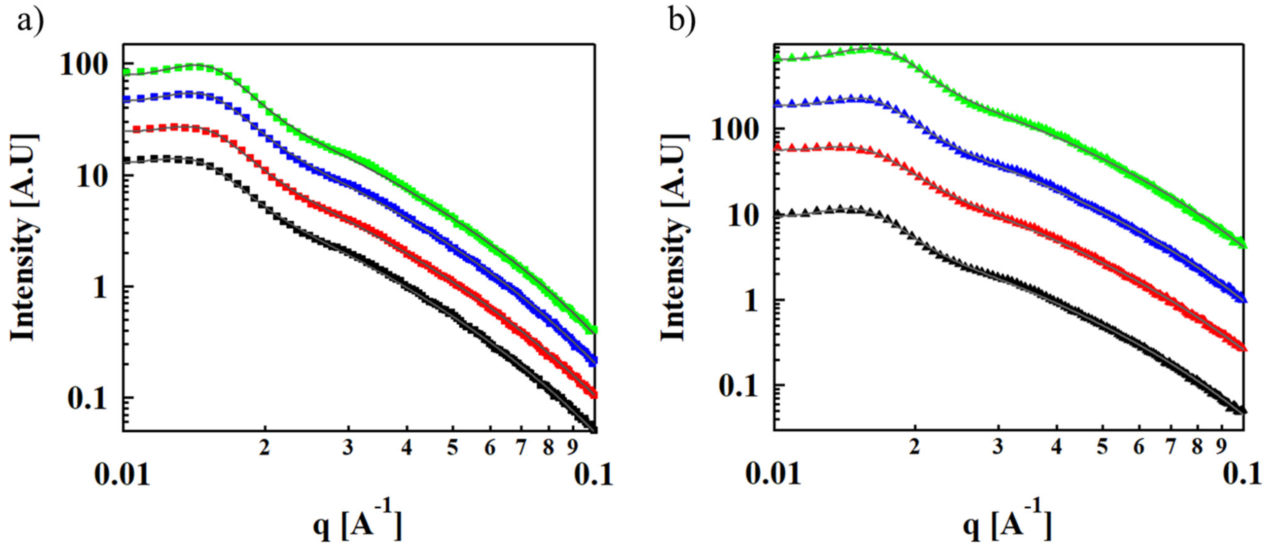

Figure S9. SAXS curves of **(a)** 6 wt% CNCs–1 wt% F127 (■) and CNCs–surfactant/polymer–SWNTCH dispersions: 6 wt% CNCs–0.5 wt% BrijS20–0.1 wt% SWNTCH (■), 6 wt% CNCs–1 wt% F108–SWNTCH (■) and 6 wt% CNCs–1 wt% F127–SWNTCH (■). **(b)** SAXS curves of 6 wt% CNCs–1 wt% F108 (▲) and 6 wt% CNCs–0.5 wt% BrijS20–0.1 wt% SWNTAP (▲), 6 wt% CNCs–1 wt% F108–SWNTAP (▲) and 6 wt% CNCs–1 wt% F127–SWNTAP (▲). The solid lines represent the fit to the stacking mode. The curves are shifted for better visualization.

Table S4. The typical thickness $\langle a \rangle$, width $\langle b \rangle$, and the inter-plate distance $\langle d_0 \rangle$ as determined from the parallelepiped stacking model.

Sample	$\langle \boldsymbol{a} \rangle$ (nm)	$\langle \boldsymbol{b} \rangle$ (nm)	$\langle \boldsymbol{d_0} \rangle$ (nm)
6 wt% CNC–0.5 wt% BrijS20–0.1 wt% SWNTAP	3.0 ± 1.3	37	37 ± 12
6 wt% CNCs–1 wt% F108–0.1 wt% SWNTAP	3.2 ± 1.3	40	36 ± 11
6 wt% CNCs–1 wt% F127–0.1 wt% SWNTAP	3.3 ± 1.1	35	35 ± 11

Table S5. The volume fraction of the nonisotropic phase ϕ_{LC}, pitch P, and data from SAXS measurements obtained from the different 6 wt% CNCs–surfactant/polymer–SWNTs suspensions.

SWNTs	Surfactant/polymer	ϕ_{LC} (vol%)	P (μm)	*SAXS*		
				q_0 (A^{-1})	q_1 (A^{-1})	q_1/q_0
SWNTCH	0.5 wt% BrijS20	49	5 ± 2	0.016	0.030	2:1
	1 wt% F108	50	5 ± 2	0.0166	0.032	2:1
	1 wt% F127	50	5 ± 2	0.017	0.033	2:1
SWNTAP	0.5 wt% BrijS20	50	4 ± 1	0.0167	0.0365	2:1
	1 wt% F108	50	6 ± 1	0.0167	0.0343	2:1
	1 wt% F127	50	6 ± 1	0.0175	0.0358	2:1

REFERNCES

1. Ribeiro, M.E.N.P.; De Moura, C.L.; Vieira, M.G.S.; Gramosa, N. V.; Chaibundit, C.; De Mattos, M.C.; Attwood, D.; Yeates, S.G.; Nixon, S.K.; Ricardo, N.M.P.S. Solubilisation capacity of Brij surfactants. *Int. J. Pharm.* **2012**, *436*, doi:10.1016/j.ijpharm.2012.07.032.

2. Mortensen, K.; Talmon, Y. Cryo-TEM and SANS Microstructural Study of Pluronic Polymer Solutions. *Macromolecules* **1995**, *28*, doi:10.1021/ma00130a016.

3. Manet, S.; elie Lecchi, A.; Imp eror-Clerc, M.; Zholobenko, V.; Durand, D.; P Oliveira, C.L.; Jan Skov Pedersen, O.; Grillo, I.; Meneau, F.; Rochas, C. Structure of Micelles of a Nonionic Block Copolymer Determined by SANS and SAXS. *J. Phys. Chem. B* **2011**, *115*, doi:10.1021/jp200212g.

4. Mao, Y.; Bleuel, M.; Lyu, Y.; Zhang, X.; Henderson, D.; Wang, H.; Briber, R.M. Phase Separation and Stack Alignment in Aqueous Cellulose Nanocrystal Suspension under Weak Magnetic Field. *Langmuir* **2018**, *34*, doi:10.1021/acs.langmuir.8b01452.

5. Mao, Y.; Liu, K.; Zhan, C.; Geng, L.; Chu, B.; Hsiao, B.S. Characterization of Nanocellulose Using Small-Angle Neutron, X-ray, and Dynamic Light Scattering Techniques. *J. Phys. Chem. B* **2017**, *121*, doi:10.1021/acs.jpcb.6b11425.
